# Relations of the Doctor to the Alcoholic Problem

**Published:** 1907-12

**Authors:** T. D. Crothers

**Affiliations:** Supt. Walnut Lodge Hospital at Hartford, Conn.


### RELATIONS OF THE DOCTOR TO THE ALCOHOLIC PROBLEM.

By T. D. Crothers, M. D., Supt. Walnut Lodge Hospital at Hartford, Conn.

---

The special purpose of this paper is to emphasize some unrecognized relations which the physician sustains to this problem, and to point out its intimate association with every day life as a literal new and unoccupied field of practice; also to urge that the alcoholic problem when studied scientifically the same as any other disease, will reveal means and measures which can be used in its prevention and curability.

The alcoholic problem presents many different aspects. One of the most startling is that seen from the statistical side. The following are some of the facts which are unquestioned, and in no way depend upon personal opinions or theories.

Studies of the mortality tables in this country and England, covering a long period of time, and made up largely from local and collective studies of cities, hospitals, insurance companies, etc., indicate that alcohol is a direct and indirect cause of from 10 to 25 per cent of all deaths. These present the highest and lowest estimates and are supported by a great variety of facts. Statistics of mental diseases and insanity indicates that alcohol is an active and contributary cause in from 15 to 50 per cent of all forms of mental diseases.

Studies of pauperism and idiocy show a remarkable agreement of authorities in the conclusion that fully 50 per cent of these forms of degeneration are traceable to this cause.

Alcohol as an active cause in criminality is apparent from the fact that in 1906 one hundred and forty-nine thousand persons were serving sentences in prisons for crime in this country, and over 50 per cent of the crimes committed while under the influence of alcohol, were due directly and indirectly to this cause. Statistics of persons arrested for petty crimes associated or following from the use of spirits, numbered over 500,000 in this

---

*Read before the Miss. Valley Med. Assn. at Columbus, Ohio, Oct. 1907.

country alone, last year. These figures refer only to persons who have become prominent by legal or hospital associations.

Beyond this, there is an unknown army of spirit and drug takers, both men and women only known to their families and occasionally to the physician, whose early death and disability give unmistakable indications of the real causes that are concealed. Reliable authorities show that over 50 per cent of all railroad accidents and disasters with automobiles and steamboats occur, and are due to the mistakes of persons under the influence of spirits. These conclusions are based on facts that are indisputable.

A view from the economic side, of losses and revolutions in business and home life, disasters of societies and failures is very startling. The observation of every physician presents many examples of revolutions, changes, mortalities, diseases and degenerations, and many of these conditions are transmitted into the next generation, or can be traced back from the generation past, and all are traceable to the free use of spirits as a beverage. Disease and degeneration that comes specifically from these causes when grouped and compared, indicate magnitude and mortality both direct and indirect far greater than that of tuberculosis, yellow fever or any of the well known epidemic diseases.

The prevalence of the disease following the use of alcohol has long been recognized by the public, and theories that it is a great moral defect to be overcome by spiritual training, education and legal measures founded on these themes have prevailed. Tremenduous efforts are being made along these lines. Thus a great political party has been formed which polled over half a million votes, societies and crusade movements are organized, innumerable laws have been proposed and passed; fines and penalties and a great variety of means and measures are urged with increasing intensity, with the one specific purpose and hope of breaking up, neutralizing and stamping out the causes and degenerations due directly to alcohol.

The alcoholic problem as a moral evil is becoming one of the great topics of church and state in every circle of society. In the light of scientific research the tides of desolution and degeneration, with frightful mortality and disease which spring from this source are ouly explainable from a study of the laws of desolution; and of cause and effect which move along exact lines from a definite origin and through the various stages of growth, development and termination.

The certain disease and death of armies of persons find no adequate explanation in the moral theories used to explain the alcoholic phenomena of today. Physicians have not taken an active part in these great temperance movements . They have accepted the usual theories of the day and considered the work outside of their own.

Where laymen and reformers have pressed the newer teachings of science to most startling conclusions, physicians have been in opposition, simply because they have not made any critical study of the subject. Often medical men are constitutionally incapable of forming new opinions, particularly when such opinions require readjustment and change of their previous views and practice. There are such men everywhere, who oppose every new movement of science, and deny that there is any progress. To them the teachings of the past are absolute and their own uncertain experience is a more accurate guide.

The alcoholic problem and its mass of scientific facts have to pass the same ordeal which greets every new truth. First the stage of indifference and denial, then a stage of partial recognition and an attempt to explain its phenomena without violating the old theories. Then the final stage of acceptance. This great problem is now in the second and third stages.

To a large class of medical men, alcohol is still a stlmulant and a good tonic, and a concentrated food, and the inebriate is a poor vice-driven weakling and the moral efforts to help him are all that can be done. To a smaller class of medical men there is a physical basis for the drink problem and possibly an element of disease, but the remedies are still moral in the first stage, with a possible physical help later. To a small number of pioneers the whole problem is a physical one governed by laws of desolution, the recognition of which will indicate the means of cure and prevention.

The newer age of sharp criticism and demand for the reasons of all theories put forward to explain the various phenomena of life has come. Questions are now asked if the theories concerning alcohol which have come down to us from the past are true. Why is it so destructive and degenerative in its effects? How can we explain the observed mental, muscular and nutrient degenerations which comes from its use, and from these theories? If we put aside all theories and take up the alcoholic problem as a mass of facts that are verifiable and apparent everywhere, one is astonished that medical men should not be teachers in this field.

The alcoholic problem comprises so many exact scientific questions which require the most accurate observation of facts and their meanings to understand them, that it would seem impossible for any but physicians and trained scientists to discover and trace the laws controling them, and point out the possibility of prevention and control. Why should laymen and moralists be forced to take up this alcoholic subject and attempt to remedy and check the maladies from on purely theoretical grounds? The only answer is, because physicians have failed. The attempt of laymen to prevent small-pox, tuberculosis, typhoid fever, dyptheria and other diseases by moral efforts would be equally absurd.

Another view indicates a very distinct field of prevention and curative medicine. The active physician of today recognizes the intimate relation of the effects of alcohol in a great variety of diseases. Thus, the man with pneumonia, who has used spirits is more likely to die. His vitality is low, his power of resistence is enfeebled, and death is the rule and recovery the exception.

Sir Wm. Broadbent said, that acute pneumonia in the alcoholic was almost always fatal. In his experience only forty persons recovered in the history of 800 cases. Practical physicians realize that the prognosis in nearly all diseases is very largely influenced by the fact of having used spirits, particularly in Brights disease, tuberculosis and other acute affections. Life insurance examiners find in the mortality lists, that the names of the diseases were only the conditions which preceded death while the real causes, alcohol and drugs, are not mentioned.

The modern surgeon always inquires concerning the habits of the patient relating to the use of alcohol, before the operation, and his prognosis is based on this history. The physicians in the medical wards of hospitals give a very guarded prognosis in all patients with an alcoholic history.

Alcohol is like syphilis, only more acute and is a degenerative agent that must be considered in the prognosis of all patients. Here it is not a question of opinion or personal prejudices; it is one of facts and a recognition of the actual conditions present. Going back a little further, the inherited defect noted in imperfect development, diseased tendencies, psychopathic weaknesses, feeble pain centers, and other conditions are all traceable to the alcoholic degenerations of parents, and these must be considered in the treatment.

Another view is still more startling. When a large number of inebriates and persons who have used spirits to excess are examined and their present condition and hereditary history are carefully gathered and compared, a remarkable uniformity appears. Thus in a certain number of cases a very large percentage have inebriate, insane and neurotic ancestors with marked constitutional defects of the brain and nervous system, also nutrition and growth, which later develope into the drink and drug craze. In others the use of alcohol dates from injury, disease, shock and definite physical conditions. Others have a history of starvation and nutrient excesses in early or later life. A certain number appear in persons with overwork, exhaustion, mental strain and neglect of normal living. The list of causes appears with such distinctness and uniformity that there can be no other questions than that of disease. If the examination is confined to the present conditions the same uniformity of symptoms appear.

The sensory integrity and nutritional balance and also the power of control is most seriously interfered with. In brief, every case is marked by distinct physical causes of which sclerosis palsies, congestion and degenerations, both local and general are common.

These facts are not apparent except from careful observation and the physician often fails to recognize them. He believes in moral causes when called to treat such persons and always fails. When he is sent for at night by families of trusted patrons, a half conscious regret and sense of impotency comes over him, that he does not understand the conditions, and the possible means for relief. If he was familar with this malady he would apply means and remedies with the same exactness as in other diseases, and the Quacks and gold cures would have no business.

The success of irregulars who by extravagant claims take advantage of the credulity of the public is evidence that the physician has failed to give the required help. All the means and measures used by the Quacks are well known to the profession and if given with scientific precision by trained men, the results would be far greater than any claimed. These pretenders use only certain concealed drugs invested in mysticism, while the scientific physician uses various remedies with other exact means particularly adapted to each individual case. In reality, the success of the specific gold cures is a most startling reflection on the neglect and failure of the medical man to occupy this field of practice; and where the physician endorses these schemes and patronizes them with a real or half faith, he advertises his failure as a scientific man. One of the specific emperics claims to have treated a half million persons for spirit and drug taking in ten years. Others claim equally large numbers. The exact number is of little moment, but the saddest fact is, that charlatans with pretention and mystery should have reaped so rich a harvest in the field of practice neglected by medical men.

There is another view with a pathetic coloring, which brings out the fact of disease and mystery. This is seen in physicians who with all their training and knowledge of physiological and psychological laws and its application to practical life should fall a victim of this disease, and become literal suicide without realizing their condition. Of course there is profound ignorance at the start with a great variety of favoring conditions, diseased impulses, and lessening of the power of resistance. Medical colleges are often responsible for this and the elaborate teaching and studies of drugs and narcotics contain little or no warning of their possible danger, except in the most informal and uncertain way. The result is that the graduate is forced to learn by experience what he should have been taught in the class room. When appealed to in private practice to help these poor victims, he can do little more than the clergyman, and it is this failure of both the recent graduate as well as the older physician to give substantial aid and treatment, that drives the poor victim into the merciless clutches of the Quack. Moral counsel and placeboes to a starved poisoned inebriate practically shuts the door and drives him into amore incurable condition.

The physical state of inebriety and the facts relating and controling it are clear and unmistakable, and indicate not only the possibility, but the certainty of the therapeutic power of means and measures for its successful cure and prevention. There is already the strongest evidence of the curability of quite a large percentage of these spirit and drug neurotics particularly when treated in the early stages. Like every other disease it has both a preventable and curable stage, especially when seen by the family physician and where the causes are recognized, and scientific means are used.

The alcoholic problem is a question of prevention as well as cure and can be done under the care and direction of the family physician. While the physiologist, laboratory men and moderate drinking critics are greatly concerned as to the exact action of alcohol on th cell and tissue, the fact of the steady growth and development of an army of alcoholic degenerates is lost sight of. All authorities admit that the action of alcohol is anaesthetic and narcotic and that its use creates a morbid impulse for more. Its special corroding effects on cell and tissue are recognized, but where its effects begin and when alcohol becomes anaesthetic in its action are questions unsettled. How far can alcohol be used as a stimulant or as a tonic; and when are these supposed actions changed, are very small questions when compared with the constantly increasing armies of poisoned and starved victims in every community. It is no wonder that the question of relief should be intensely agitated in societies, homes, churches and be a subject of discussion in all circles. Without accurate knowledge of the exact causes and conditions, all efforts of the laymen and others must necessarily be uncertain and fail to reach and overcome the malady.

There is a practical side to the problem which we must recognize. At present the medical man who would be indifferent to the presence of contageous diseases and refuse to give aid and assistance in their cure and prevention, would most naturally be condemned. In much the same way the physician who neglects to study this problem and the causes and conditions which govern its growth and development, in every circle of life, is as positively reprehensible.

First, because he is a physician and these are questions of disease and degeneration concerning the operation of physical laws and physical forces with which he is constantly dealing.

Second, because he is trained to observe physical facts and study their meanings. He is able to point out the growth, development, direction physiological forces and environmental conditions which influence human life; lastly because he is a citizen and an educated man and is concerned and deeply interested with every phenomena which relates to the welfare of the race. The medical man is best fitted and trained to understand the alcoholic problem from an exact and scientific point of view. He is practically a student of physiology, psychology and sociology and is constantly judging and noting the various influences that enter into the evolution and desolution of humanity. Therefore the alcoholic problem is a province in which it is his duty to study, teach and direct how it can be overcome and not stand off coldly indifferent to the tremenduous efforts by laymen and others to understand and remedy this malady. It is his duty, not to condemn the efforts of others, but rather to lead them and direct them into exact lines. He should be a temperance man in the broadest sense of this word, and his personal life and example should confirm and strengthen his teachings. The physician who uses alcohol to excess is not only a diseased man, but deplorably ignorant of the whole subject and his conduct proclaims to his associates incapacity to understand or control himself.

The physician who defends the moderate use of spirits as a beverage and puts in practice his theory has lost his way as a medical man and is sadly belated in the march of science.

Every year the magnitude of the injuries and losses which come directly from the use of spirits, brings a greater responsibility to the physician and more imperative reason for his direction in the efforts to neutralize and prevent these injuries.

The late Anti-Alcoholic Congress held in Stockholm,Sweden, last August brought out this fact very clearly. Fifteen hundred delegates from all over the world gathered and for five days discussed the different phases of the alcoholic problem. Less than one hundred physicians were present and only about thirty took an active part. The Congress as a whole was a great protest against medical indifference and failure to take up this problem and teach the facts. Laymen, clergymen, philanthropists and others presented startling arrays of facts and conclusions with the same appealing inquiry to physicians,—what can be done and what means can be used for the cure and prevention? How can this evil be stamped out? A number of physicians including some of the great medical teachers of Europe, read papers pointing out lines and giving indications for more practical and exhaustive studies. These papers were not theoretical, but scientific deductions and conclusions from the facts at present considered reliable. The anaesthetic action of alcohol and its active and most intimate cause of disease was reaffirmed and the necessity urged for more exact studies which would indicate the means of prevention and cure was made very prominent.

This Congress will meet again in London in 1909, when the facts concerning physicians and their relations to this great problem will come into great prominence. The medical profession parti cularly in this country, are brought face to face with a problem which they must solve. Public opinion demands in no uncertain terms that the questions of alcohol and its influence be settled by the physician. Temperance crusades and great revival efforts with their profound psychical influence, turn to the medical men for help and counsel. Every effort by laymen with moral and legal means to understand and break up this malady is a reflection and distinct intimation of the failure of medical men to understand this subject. If the physician would study the alcoholic problem from the scientific side, the Quack and the Gold Curers would disappear and the great temperance agitations would take on a practical scientific aspect under the direction of physicians and the great problem would be made clear.

The present attitude of indifference and silent opposition to the enthusiastic struggles of those who are trying to educate public sentiment and neutralize and overcome the losses from this source, by the profession is most irrational, unscientific and unaccountable. The physician should be the practical and logical authority as to the means and remedies which follow from the use of alcohol in every community, and his failure to do this is unconsciously to forfeit his respect as a scientific man.

The problem is a living vital one, not of ethics or morals, but of disease and death; the facts are available, and why they have not been recognized before is a matter of profound astonishment. If the medical profession would take up this alcohol problem and study the facts in their true light, there would be no disputed questions of license, prohibition, control and punishment of the victims.

The American Society for the study of Alcohol and other

Narcotics, has this as its one purpose and object to take the whole subject out of the hands of laymen and irregulars and bring it into the realm of science. This is the imperative need of the hour, and this is the new land for medical study at our very door white for he harvest with possibilities beyond any scientific conception of the present.

The following conclusions should be recognized by every medical man:—

First, every advance in the scientific study of disease brings into increasing prominence the physical laws and causes of the alcoholic problem ,showing that the facts and conditions of this disease can be traced and verified in every community and society.

Second, the alcoholic problem as understood at present is based on theories which utterly fail to explain the phenomena and conditions which follow. Efforts to apply these theories in practical treatment, increase the degenerations which they seek to prevent. This is strikingly confirmed in the legal measures to prevent and cure this malady.

Third, the medical profession are particularly fitted by education and study to understand the physical laws and causes relating to the alcoholic problem and the phenomena which follows and should be the leaders and teachers in all efforts for its cure and prevention.

Fourth, the failure of the medical profession to take up this subject has opened the door for Quacks and emperics, claiming the most marvelous discoveries and results in cures, and also great crusade temperance movements conducted by men without knowledge and by victims themselves, who try to teach the public the means for cure and prevention.

Fifth, it is this remarkable indifference of medical men that has permitted public sentiment to endorse and sustain a great variety of measures for cure based on wrong theories which will disappear when the subject is studied from a medical standpoint.

Lastly,this is the great new land of medicine awaiting development and discovery; a land where the facts when known will clearly point out the means for cure and prevention, which when applied will both literally and practically stamp out this great scourge of modern civilization.

